# Monkeypox: An Emerging Global Public Health Emergency

**DOI:** 10.3390/life12101590

**Published:** 2022-10-12

**Authors:** Madhan Jeyaraman, Preethi Selvaraj, Manjunatha Budihal Halesh, Naveen Jeyaraman, Arulkumar Nallakumarasamy, Manu Gupta, Nicola Maffulli, Ashim Gupta

**Affiliations:** 1 Department of Orthopaedics, Faculty of Medicine, Sri Lalithambigai Medical College and Hospital, Dr. MGR Educational and Research Institute, Chennai 600095, Tamil Nadu, India; 2 South Texas Orthopaedic Research Institute (STORI Inc.), Laredo, TX 78045, USA; 3 Department of Community Medicine, Faculty of Medicine, Sri Lalithambigai Medical College and Hospital, Dr. MGR Educational and Research Institute, Chennai 600095, Tamil Nadu, India; 4 Department of Emergency Medicine, SDM Medical College, Dharwad 600095, Karnataka, India; 5 Department of Orthopaedics, Atlas Hospitals, Tiruchirappalli 620002, Tamil Nadu, India; 6 Department of Orthopaedics, All India Institute of Medical Sciences, Bhubaneswar 751019, Odisha, India; 7 Polar Aesthetics Dental & Cosmetic Center, Noida 201301, Uttar Pradesh, India; 8 Department of Musculoskeletal Disorders, School of Medicine and Surgery, University of Salerno, 84084 Fisciano, Italy; 9 San Giovanni di Dio e Ruggi D’Aragona Hospital “Clinica Ortopedica” Department, Hospital of Salerno, 84124 Salerno, Italy; 10 Centre for Sports and Exercise Medicine, Barts and the London School of Medicine and Dentistry, Queen Mary University of London, London E1 4DG, UK; 11 School of Pharmacy and Bioengineering, Keele University School of Medicine, Stoke on Trent ST5 5BG, UK; 12 BioIntegrate, Lawrenceville, GA 30043, USA; 13 Future Biologics, Lawrenceville, GA 30043, USA; 14 Regenerative Orthopaedics, Noida 201301, Uttar Pradesh, India

**Keywords:** monkeypox, orthopoxvirus, public health emergency, World Health Organization

## Abstract

The virus causing monkeypox, a rare zoonotic viral disease, belongs to the Poxviridae family and the Orthopoxvirus genus. On 23 July 2022, the World Health Organization (WHO) declared the monkeypox outbreak as a Public Health Emergency of International Concern (PHEIC). From May to July 2022, a multi-country outbreak of monkeypox was reported in both endemic and non-endemic regions. Major goals of managing monkeypox are to identify the suspected cases, detect generic orthopoxvirus DNA at a state or commercial laboratory, and establish the Centers for Disease Control and Prevention real-time polymerase chain reaction testing. Currently, there are no approved treatments for monkeypox virus infection. However, a variety of antiviral medications originally designed for the treatment of smallpox and other viral infections could be considered. Pre-exposure prophylaxis for laboratory and health care employees and post-exposure prophylaxis for individuals with high-risk or intermediate-risk exposures are to be considered. The CDC Emergency Operations Center is available for advice on the appropriate use of medical countermeasures, and can help in obtaining antiviral drugs and vaccines from the National Strategic Stockpile. This review gives an overview of the global scenario, clinical presentation, and management of monkeypox in the light of a global public health emergency.

## 1. Introduction

**Figure 1:**
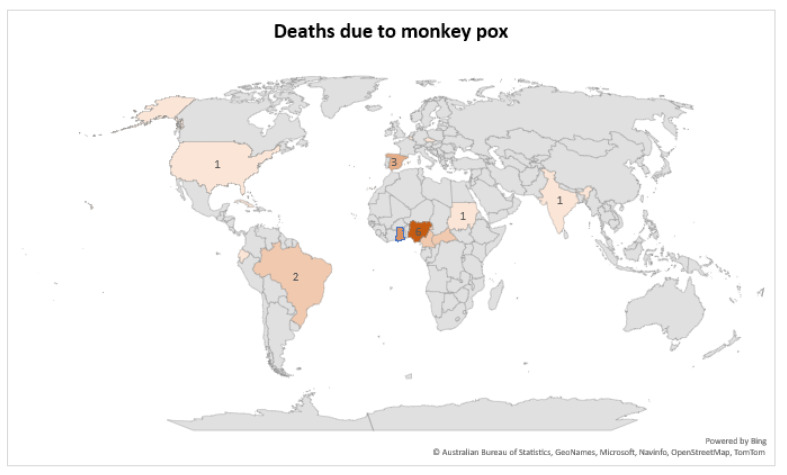
Global distribution of deaths due to monkey pox (created on biorender.com).

The monkeypox virus, a member of the Poxviridae family and the Orthopoxvirus genus, causes monkeypox, a rare zoonotic viral illness [1], with mortality rates ranging from 3% to 6% in African nations where the disease is widespread. Deaths caused by monkeypox have been extremely uncommon, with only 15 cases of fatalities reported worldwide. Deaths have been reported from countries in West and Central Africa, where the virus is endemic, but also from United States, Spain, and Brazil. Historically, these non-African countries have not been susceptible to the virus’s spread [2] (Figure 1). A multi-country outbreak with atypical clinical symptoms has been reported in non-endemic nations, with many cases diagnosed in men who have sex with other men [2]. Since 2022, an outbreak of monkeypox has been reported in various nations, and a frequently updated situation summary of monkeypox cases in the United States and Europe are accessible on the Centers for Disease Control and Prevention (CDC) website [3]. The World Health Organization (WHO) also gives monthly updates on the outbreak, which has spread to many countries. Traveling to high-risk nations or interacting with high-risk animals, animal products, or people could have resulted in this exposure. A diagnosis of monkeypox requires laboratory detection of the monkeypox virus, and a review of pertinent epidemiologic risk factors and clinical signs [4,5]. The characteristic symptoms of monkeypox include a rash, fever, chills, headache, myalgia, arthralgia, lymphadenopathy, and a clinical course similar to but less severe than smallpox [1,4,6]. The rash is almost always present in monkeypox, with skin lesions that are deep-seated, well-demarcated, and umbilicated; early lesions in mucosal regions (oral, vaginal, perianal) have been common in the 2022 monkeypox patients [5]. 

Diagnostic testing for monkeypox should be undertaken in two steps, in conjunction with public health authorities. The first stage is to identify the presence of generic orthopoxvirus DNA at a state or commercial laboratory, and the second step is to have the Centers for Disease Control and Prevention conduct confirmatory real-time polymerase chain reaction testing for monkeypox (CDC) [7]. Patients with severe disease, those at high risk of developing severe disease, or those with infections involving high-risk anatomic areas may be candidates for antiviral treatment [8]. Most monkeypox patients have a mild version of the disease and can be managed with supportive treatment [9]. There are currently no approved treatments for monkeypox virus infection; however, several antiviral drugs originally designed for the treatment of smallpox and other viral infections are available [10].

To limit the likelihood of being exposed to infectious agents and contracting an infection, measures must be implemented in both clinical and home settings. A smallpox vaccination as post-exposure prophylaxis is advised for high-risk exposures and can be considered for intermediate-risk exposures. Exposed individuals should be checked for symptoms and their exposure risk assessed [11]. Vaccination is suggested as pre-exposure prophylaxis for some laboratory and health care employees who are at continued risk of occupational exposure, and it can also be administered as post-exposure prophylaxis for persons who have had high-risk or intermediate-risk exposures [12]. Individuals who have recently been exposed to monkeypox are being offered vaccinations in some public health jurisdictions as part of a broader approach to post-exposure prophylaxis in preparation for a major outbreak [13]. This is occurring even though the individuals have had no documented exposure to someone who has been diagnosed with monkeypox. The CDC Emergency Operations Center is available for advice on the proper use of medical countermeasures for monkeypox, and it can help to obtain antiviral drugs and vaccines from the National Strategic Stockpile [14,15]. 

## 2. Global Scenario

The first case of human monkeypox was reported in 1970 in the Democratic Republic of the Congo, [16]. A 9-month-old boy developed the disease, which occurred in a region of the country where smallpox had been eradicated in 1968 [16]. Since then, the vast majority of cases have been recorded from the Congo Basin’s rural and rainforest regions, mainly the Democratic Republic of the Congo, with human cases progressively being reported from across Central and West Africa [17]. 

Human cases of monkeypox have been documented in 11 different African countries since 1970 [18] (Table 1). Benin, Cameroon, the Central African Republic, the Democratic Republic of the Congo, Gabon, Cote d’Ivoire, Liberia, Nigeria, the Republic of the Congo, Sierra Leone, and South Sudan are among these countries [18]. It is unknown how severely monkeypox can damage an individual. For example, the Democratic Republic of the Congo saw an outbreak with an unusually high attack rate but a low case fatality ratio in 1996–1997 [19]. The discovery of a concurrent epidemic of chickenpox (produced by the varicella virus, which is not an orthopoxvirus) and monkeypox may explain real or perceived variations in the disease’s transmission dynamics. Nigeria has been coping with a large outbreak since 2017, with over 500 suspected cases, over 200 confirmed cases, and an estimated case fatality ratio of 3% [20]. Cases continue to be reported up to the present day. 

Monkeypox affects not only countries in West and Central Africa but also the rest of the world [21], and it is regarded as a critical public health concern on a global basis. In 2003, the United States of America had the first monkeypox outbreak outside of Africa [22]. Contact with sick prairie dogs raised as pets was linked to this outbreak. These domesticated animals were housed in the same enclosure as dormice and Gambian pouched rats that had been imported from Ghana. More than 70 cases of monkeypox were diagnosed in the United States as a result of this outbreak [23]. Monkeypox was detected in Israel in September 2018; the United Kingdom in September 2018, December 2019, May 2021, and May 2022; Singapore in May 2019; and the United States of America in July and November 2021 [24]. All these incidents occurred after travelers had returned from Nigeria. Multiple cases of monkeypox were discovered in several nations in which the disease was not endemic in May 2022 [2]. Research efforts are presently underway to better understand the epidemiology, sources of illness, and patterns of transmission. 

Additionally, since the start of the monkeypox outbreak and as of 6 September 2022, 18,844 confirmed cases of monkeypox (MPX) have been reported from 29 EU/EEA countries. In total, 47 cases have been reported from three Western Balkan countries and Turkey. The five countries reporting most cases since the start of the outbreak are Spain (6749), France (3645), Germany (3505), Netherlands (1172) and Portugal (789). Two deaths have been reported from Spain in July 2022, and one death from Belgium. Recent health emergencies in the European Region have highlighted Risk Communication and Community Engagement (RCCE) as a core public health intervention which contributes to emergency response [25]. 

According to the WHO, this is the first time that chains of transmission have been identified in Europe that have no known epidemiological links to West or Central Africa in the current run of epidemics [26]. The WHO has confirmed that the global number of cases of monkeypox has risen to over 6000, with the bulk occurring in European countries [27]. There have been four confirmed instances of the monkeypox virus in India as of July 2022 [28]. The WHO has declared the monkeypox virus to be a public health emergency of international concern and issued the highest degree of alert (PHEIC) [29]. We report the present recommendations from the CDC in the USA, and we stress that similar guidelines have been issued by the European Centre for Disease Prevention and Control [26]. 

## 3. Classification

### 3.1. CDC Case Definitions

#### 3.1.1. Suspect Case

- A new rash resembling monkeypox or
- meets one epidemiologic requirement within 21 days of the onset of symptoms and has a strong clinical suspicion of having monkeypox.

#### 3.1.2. Probable Case

- Criteria for a suspect case have been met, and there are no known recent cases of exposure to the orthopoxvirus (such as through vaccination against smallpox).
- Orthopoxvirus presence confirmed through immunohistochemistry, electron microscopy, polymerase chain reaction testing of a clinical specimen, or
- anti-orthopoxvirus IgM antibody detection between 4 and 56 days after the start of the rash.
- According to the CDC, all confirmed cases of orthopoxvirus infection are assumed to be cases of monkeypox until proven otherwise for the ongoing 2022 outbreak.

#### 3.1.3. Confirmed Case

- Criteria were met for a possible case, and one of the following indicated a monkeypox infection:
  ⚬ The next-generation sequencing of a clinical specimen or the polymerase chain reaction for the detection of monkeypox virus DNA.
  ⚬ Isolation of the monkeypox virus from a clinical sample for culture [30,31].

### 3.2. WHO Case Definitions for the Current Multi-Country Monkeypox Outbreak Event (2022) Are Slightly Different 

#### 3.2.1. Suspect Case

- Unexplained acute rash in a person of any age in a monkeypox-nonendemic country and
- One or more of the following:
  ⚬ Headache.
  ⚬ Acute onset of fever.
  ⚬ Lymphadenopathy.
  ⚬ Myalgia.
  ⚬ Back pain.
  ⚬ Profound weakness.
- Additionally, other common causes of acute rash deemed incompatible with the clinical presentation.

#### 3.2.2. Probable Case

- Suspected case criteria met and at least one of the following:
- Within 21 days of the onset of symptoms, a confirmed epidemiologic link to a probable or confirmed case of monkeypox through direct physical contact, face-to-face exposure, or contact with contaminated objects.
- Travel within 21 days of the onset of symptoms to a nation where monkeypox is endemic.
- Within 21 days of the onset of symptoms, multiple or anonymous sexual partners, positive orthopoxvirus serology, and hospitalization for this illness.

#### 3.2.3. Confirmed Case

- Criteria for a suspected or probable case have been met, and monkeypox virus DNA has been confirmed in the lab using real-time PCR or sequencing [32,33].

## 4. Pathogenesis

### 4.1. Causes

After the eradication of smallpox, the monkeypox virus has been the most prevalent orthopox virus to infect humans [34,35]. Despite the name, mice and other small animals are far more frequently implicated than monkeys or other primates. The natural reservoir of the monkeypox virus is unknown [23,30,36]. The monkeypox virus has two separate genetic subgroups (clades): West African and Central African [20,36]. The present outbreak (2022) seems to be produced by the West African clade, which is associated with milder disease [37,38]. 

The virus is transferred through contact with an infected person or animal, as well as other materials contaminated with the virus. Viruses are believed to enter the body through wounds, mucosal membranes, or the respiratory system. Animal bites or scratches, meat processing, direct and indirect contact with contaminated animal debris, and other factors can all contribute to the spread of this disease from animals (e.g., bedding). The main modes of human-to-human transmission are large respiratory droplets, direct or indirect contact with body fluids, lesion material, and contaminated surfaces or items (e.g., clothing, bedding, linens). Vertical transfer from the mother to the unborn child via the placenta is possible during pregnancy. Although DNA from the monkeypox virus has been discovered in seminal fluids, it is presently uncertain whether sperm and vaginal secretions can transfer the infection. Close or intimate physical contact can aid in the spread of pathogens through respiratory and contact routes [1,39,40,41]. 

#### 4.1.1. Agent

The monkeypox virus (MPXV) is a double-stranded DNA virus with an envelope and is a member of the Orthopoxvirus genus in the Pox viridae family [1].

#### 4.1.2. Host

The natural reservoir remains undiscovered. However, some rodents, including dormice, rope squirrels, tree squirrels, Gambian pouched rats, and non-human primates, are inherently vulnerable to the monkeypox virus [23,30,42,43].

#### 4.1.3. Incubation Period

Monkeypox typically manifests its symptoms 6 to 13 days after infection, but it can take between 5 and 21 days [23,30,42,43].

#### 4.1.4. Period of Communicability

This period is 1–2 days before the rash becomes better and lasts until all the scabs have fallen off [23,30,42,43].

### 4.2. Risk Factors and/or Associations

#### 4.2.1. Age

Cases of the 2022 outbreak typically involve younger people. As such, 79 percent of cases around the world involve patients between the ages of 18 and 44. 

#### 4.2.2. Sex

Over 99.5% of all 2022 outbreak cases in the world with data on sex have been men [24,31]. 

In the present 2022 outbreak, patients tend to be younger. The median age of patients worldwide is 36 years; male patients aged 18 to 44 years account or 77.9% of cases [9]. For the 2022 outbreak cases with available sex data, over 98% of cases worldwide have been males. According to the ethnicity/race data reported to CDC, 41% of patients are White, 26% are Black, and 26% are Hispanic; data are missing for many cases 40

Sexual activity, especially male-to-male sexual contact was reported [15,17,41,42]. Most cases in the ongoing 2022 outbreak worldwide, including those in the United States, have been in men who identify as gay or bisexual or in other men who have sex with men. A history of male-to-male sexual contact was self-reported in 98.4% of male patients in US cases for which such data was available, per CDC data [14]. According to the WHO data, among cases worldwide with reported sexual orientation, 95.8% of patients identified as gay or bisexual or other men who have sex with men; a sexual encounter was implicated in 91% of all transmission events [27].

A large international case series for the 2022 outbreak, reporting on 528 cases in 16 countries, similarly found that 98% of cases were in gay or bisexual men, with sexual activity implicated in transmission in 95% of persons with infection [34]. History of multiple casual sexual encounters or multiple sexual partners further increases risk of exposure and infection [32]. Monkeypox is not considered a sexually transmitted infection in the traditional sense, but it can be spread via close skin to skin contact during sexual activity, including kissing, touching, oral sex, and penetrative sex, and by shared bedding, linens, and clothing [39]. Concomitant sexually transmitted infections were reported in 29% of patients diagnosed with monkeypox in a recent large, international case series [34]. Among 2022 outbreak cases with reported HIV status, 45% of cases worldwide have been positive for HIV; rates in individual countries have ranged from 28% to 51% [27,43]. Health care workers are at risk of monkeypox virus entry via their own unprotected skin or mucous membranes, through actions such as:

▪ Touching a patient’s skin, skin lesions, or bodily fluids;
▪ Touching contaminated materials such as linens or clothing;
▪ Allowing their own unprotected clothing to touch patient skin, skin lesions, bodily fluids, or contaminated materials such as linens or clothing;
▪ Being inside a patient’s room or near a patient during aerosol-generating procedures
▪ Close, prolonged presence near a patient;
▪ Personal and household contacts are also at risk of monkeypox infection via direct and indirect contact with monkeypox lesions, lesion material, respiratory secretions, other bodily fluids, and contaminated surfaces or materials (e.g., dishes, utensils, clothing, bedding, linens).

### 4.3. Clinical Presentation

A patient with relevant clinical signs, including rash, and suspected monkeypox virus exposure through travel to high-risk countries or contact with high-risk animals, animal products, or people should be investigated for monkeypox [1,5,8]. 

### 4.4. Clinical History

The incubation period lasts between one and two weeks, and the patients may not show any symptoms. Patients may be contagious during this time; the initial prodromal symptoms are nonspecific and include fever, malaise, headache, weakness, and lymphadenopathy. Prodromal symptoms are frequently mild or nonexistent in cases of the 2022 outbreak, despite being frequent in cases of classic endemic monkeypox. The presence of a rash within 5 days of the onset of the illness should point towards conditions other than monkeypox, as the rash can appear as the first symptom, without fever or any other prodromal symptoms, but it usually appears several days after a fever. Initial rash in mucosal regions (oral, genital, perianal) is more common in cases of the 2022 outbreak; and initial rash on the face or in the oral cavity is frequently described in classic endemic monkeypox. On any given body part (such as the face or genitalia), classic endemic monkeypox lesions usually appear and progress simultaneously; however, asynchronous evolution has been observed in cases associated with the 2022 outbreak. Before scabbing over and healing, lesions go through macular, papular, vesicular, and pustular morphologies. Until they heal, lesions are said to hurt. Perianal lesions may cause anorectal pain, tenesmus, and rectal bleeding. Patients are infectious until all lesions have healed, scabbed over, and been covered by a new, healthy layer of skin. The average length of the illness is two to four weeks [1,43].

## 5. Diagnosis

The diagnosis of monkeypox is made by laboratory detection of the monkeypox virus in the presence of the necessary epidemiologic risk factors and clinical symptoms [43,44,45,46,47].

According to the Centers for Disease Control and Prevention (CDC), all confirmed orthopox cases for the ongoing monkeypox outbreak in 2022 (such as a suspect case with an orthopoxvirus infection confirmed by a state laboratory or qualified commercial laboratory) are assumed to be monkeypox until proven otherwise [30]. Additionally, the WHO has released interim recommendations for monkeypox surveillance, reporting, case investigation, and contact tracing, for use worldwide [48]. 

Specific diagnostic tests: For assistance with diagnosis, possible cases of monkeypox should be reported to the appropriate local and state public health authorities; CDC consultation is also available through the Emergency Operations Center (telephone, 1-770-488-7100) [30]. According to the CDC, all confirmed orthopox cases for the ongoing outbreak event in 2022 are assumed to be monkeypox until proven otherwise. The CDC conducts real-time polymerase chain reaction confirmation testing for monkeypox, which looks for the presence of monkeypox virus DNA. Other diagnostic procedures for monkeypox and orthopox have included viral culture and isolation, electron microscopy, immunohistochemistry, and testing for orthopox antibodies or antigens; however, these procedures are no longer advised [30]. If a patient has epidemiologic risk factors as specified in the CDC case definition and either a classic monkeypox rash or a potential monkeypox rash is present, the CDC advises collecting swabs for testing (i.e., contact with someone with a similar rash or confirmed monkeypox diagnosis, close contact with people in a social network with monkeypox activity, history of recent international travel to the country with confirmed cases). Specimens should be collected while wearing the proper personal protective equipment, such as a N95-comparable or higher-level respirator, gown, gloves, and eye protection [49]. Lesion material is typically used for generic orthopox testing at Laboratory Response Network labs and authorized commercial labs; specific requirements for state, commercial, and CDC specimen collection may vary [30]. Before sending samples to the CDC, consultation with the state epidemiologist, state health laboratory, and CDC is necessary [30]. 

Complete information for specimen submission to CDC is available on the CDC website [30]:

- Swabs of the lesion’s surface or fluid are acceptable when dry.
- Two swabs should be taken from each lesion, and lesions from various body regions or with various morphological characteristics should be sampled in particular.
- Within an hour of collection, samples should be frozen (at 20 degrees Celsius or lower); frozen samples can be kept for up to 60 days.
- You should not include any more transport media.
- For CDC testing, the turnaround time is 5 days.

Internationally applicable interim guidelines for monkeypox virus laboratory testing have also been released by WHO [50]. 

## 6. Management

Many monkeypox patients have a mild, self-limiting disease that can be controlled with supportive care only [11,12]. Potential management strategies may include the following, depending on the systems involved and the specific manifestations or complications of the disease:

- Respiratory tract: bronchodilators, antibiotics for secondary respiratory infections, chest physical therapy, nebulizer treatments, suctioning, incentive spirometry, bronchoscopy, noninvasive ventilation, and intubation/ventilation.
- Sepsis: IV fluids, vasopressors, antibiotics, and extra oxygen.
- Oral lesions: oral hygiene and topical or oral analgesics.
- Vomiting and diarrhea: Hydration via oral or IV as well as antiemetic and antidiarrheal medications.
- Fever: Antipyretic medications and external cooling techniques.
- Skin compromise: Cleaning, moist dressings, topical antibiotics, surgical debridement, and skin grafts.
- Secondary skin infection: Antibiotics, wound incision and drainage, and advanced wound management.
- Lymphadenopathy and inflammation: Anti-inflammatory and analgesic drugs.
- Ocular infection: Slit lamp examination, comprehensive ophthalmic evaluation, and use of ophthalmic antibiotics, antivirals, and corticosteroids.

There are currently no approved treatments for monkeypox virus infection, but state health authorities may request and obtain several antiviral medications from the Strategic National Stockpile upon request after consulting with the CDC [11,51,52]. The lack of efficacy data for these medications is caused by the impossibility of conducting human smallpox experimentation.

(a) Tecovirimat: Endorsed for the treatment of human smallpox in adults and children 3 kg or more. Available as both capsules and injections, Tecovirimat demonstrated effectiveness in both in vitro and in vivo animal studies against a wide variety of orthopoxviruses. The CDC has a protocol known as EA-IND (expanded access-investigational new drug) that permits use in treating monkeypox during an outbreak. In patients with severe renal impairment, injection is not recommended [53,54,55,56].
(b) Cidofovir: Has been used to treat other viral infections since being initially approved for the treatment of cytomegalovirus in AIDS patients. The EA-IND protocol is available from the CDC, and it can be used to treat monkeypox during an outbreak. To minimize the risk of nephrotoxicity, oral probenecid is taken with each cidofovir dose. Probenecid Oral Tablet; Adults: 2 g administered orally 3 h before each cidofovir infusion, then 1 g administered orally 2 and 8 h after the infusion. For neutropenia and renal impairment, there are BLACK BOX WARNINGS. Patients receiving potentially nephrotoxic medications, those who have severe cidofovir hypersensitivity, and those who have severe sensitivity to probenecid or other sulfa-containing drugs should not take this medication [11,12,57].
(c) VIGIV (vaccinia immune globulin intravenous): Approved for the management of vaccine-related side effects. The EA-IND protocol is available from the CDC, and it can be used to treat monkeypox during an outbreak. only for IV use. Adults: 6,000 units/kg/dose IV as soon as symptoms arise; repeat dosing may be considered depending on symptom severity and response to treatment; and 9000 units/kg/dose in patients who do not react to the initial dosage. For interactions with glucose monitoring systems, see BLACK BOX WARNING. During VIGIV therapy, blood sugar should be checked using a glucose-specific technique because VIGIV contains maltose, which can cause falsely elevated glucose readings on some glucose monitoring devices. Patients with a history of anaphylaxis or another severe reaction to IV immune globulin, as well as IgA-deficient individuals with anti-IgA antibodies and a history of IgA hypersensitivity, should not receive this medication [58,59].
(d) Brincidofovir: Approved for the treatment of adult, pediatric, and neonatal cases of human smallpox. Monkeypox treatment is made easier by the EA-IND protocol that the CDC is currently developing. Brincidofovir is not yet accessible from the Strategic National Stockpile. Although the CDC does not presently have an EA-IND protocol for brincidofovir for monkeypox, one is being developed to facilitate its usage. While fasting, administer Brincidofovir oral suspension; newborns: 6 mg/kg/dose administered orally once a week for two weeks (on days 1 and 8). BLACK BOX WARNING: Prolonged use may increase the risk of mortality due to the drug overdosage. The prescribing information contains no contraindications [8,10,52].

## 7. Prevention

It is advisable to avoid close, unprotected contact, including sexual contact, with anyone who has a rash resembling monkeypox. In addition, it is also advisable to maintain distance from potentially infected animals, animal products, or anything that came into contact with sick people or animals. Infected patients should also be kept apart. It is also advisable to maintain suitable hand hygiene and use of personal protective equipment (PPE) while caring for patients or gathering samples for diagnosis [9,60]. 

### 7.1. Infection Prevention in Medical Facilities

The CDC refers to the 2007 guidelines for preventing the spread of infectious agents in health care settings when discussing the prevention of monkeypox transmission in those settings. 

Standard precautions should be taken with patients who have monkeypox infection: they should be kept in single-person rooms. Special air handling is not required unless high-risk procedures are carried out. Only visitors who are required for the care and well-being of the patient should be allowed. Airborne isolation rooms should be used for intubation, extubation, and any other high-risk procedures that could potentially spread oral secretions. When entering the patient’s room, medical personnel should be wearing personal protective equipment such as a gown, gloves, eye protection, and a respirator with an N95-comparable or higher level of protection. The management of waste should be performed according to the rules and regulations for hazardous materials. Observe the recommended standard practices for environmental infection control when handling soiled laundry. Utilizing hospital-grade disinfectants with an emerging viral pathogen claim should be used for cleaning and disinfection. The use of dry dusting, sweeping, and vacuuming should be avoided in favor of wet cleaning techniques. Infection control precautions should be kept in place until all lesions have separated, healed, and produced a new layer of healthy skin, or until the public health authorities have given their approval [29,61,62]. 

### 7.2. Preventing Infections in Domestic Settings 

#### 7.2.1. Isolation and Personal Protective Equipment

Patients not requiring hospitalization should be isolated at home and any contact with other household members or pets should be avoided. No visitors should be allowed during the isolation period as well. Such patients should also not leave their house unless it is an emergency or they require medical attention. In cases where close contact is essential or cannot be avoided, both patients and other household members should use PPE. A separate bathroom should be utilized by the patients, if possible. Proper PPE should be used while performing wound care or handling linens and laundry either by the patient themselves or other household members. It is better to cover skin lesions, and wear long sleeves, long pants/trousers, and gloves as often as possible. Patients should also avoid wearing contact lenses and shaving any rash-affected areas to help limit spread of infection [63,64,65].

#### 7.2.2. Hand Hygiene and Household Disinfection

It is critical to wash potentially contaminated personal objects such as clothes, linens, towels, plates, and utensils and to prevent sharing them. Countertops and light switches that are used frequently should be cleaned and disinfected regularly. It is critical for the patients to wash their hands with soap and water or an alcohol-based hand rub after coming into contact with any rash-causing chemicals or potentially contaminated objects or surfaces. If another restroom is not available, potentially contaminated surfaces must be cleaned and sanitized after each use of the communal area. The CDC has issued thorough interim recommendations for cleaning dwellings when monkeypox is suspected/proven. Infection control procedures should be maintained until all lesions have scabbed off, separated, and healed with a fresh layer of intact skin, or until cleared by public health authorities [63,64,65]. 

### 7.3. Management Recommendations Depend on the Degree of Exposure

#### 7.3.1. High Risk

Symptoms should be monitored, and ACAM2000 (Sanofi Pasteur Biologics, Cambridge, MA, USA) or the Jynneos vaccine (Bavarian Nordic, Denmark) should be used for post-exposure prophylaxis. Being in the patient’s room or within 6 feet of a patient during aerosol-generating procedures without wearing an N95-comparable or higher-level respirator and eye protection, lesions, bodily fluids, or contaminated materials from a patient, or other exposures deemed at this level by public health authorities [11,66].

#### 7.3.2. Intermediate Risk

Individual needs for post-exposure prophylaxis can be determined by monitoring symptoms. Being at least 6 feet away from an unmasked patient for three hours or more without wearing a surgical mask or sleeves or other clothing coming into contact with the patient’s skin, lesions, bodily fluids, contaminated materials, or other exposures determined by public health authorities to be at this level [11,66].

#### 7.3.3. Low/Uncertain Risk

It is recommended that affected individuals monitor their symptoms; post-exposure prophylaxis is not required. Low or unknown risk exposures include: entering the patient’s room without eye protection, regardless of how long you stay/Visitors must wear a surgical mask, a gown, gloves, and eye protection when entering the patient’s room. Being within 6 feet of an uncovered patient for less than 3 h without wearing at least a surgical mask or other exposures assessed by public health authorities to be at this level [11,66].

#### 7.3.4. No Risk

Post-exposure prophylaxis and symptom monitoring are not required. These exposures do not match the requirements for the other categories, according to public health authorities [11,66]. 

## 8. Vaccination

### 8.1. Pre-Exposure Prophylaxis

Although there are no vaccination efficacy data for the current outbreak, the efficacy of smallpox vaccines against monkeypox is anticipated to be at least 85% based on immunogenicity and animal testing data [67]. Jynneos is a vaccine approved for both monkeypox and smallpox in the United States [68,69]. ACAM2000 is another vaccine licensed for smallpox prevention but also effective for monkeypox prevention [30]. The CDC Drug Service can deliver vaccines for pre-exposure prophylactic immunization after speaking with the CDC Emergency Operations Center (telephone, 1-770-488-7100) [70]. However, some individuals, such as those with severe immunocompromission, may experience serious consequences as a result of vaccination. Vaccines are generally well tolerated, with only mild side effects. 

### 8.2. Post-Exposure Prophylaxis

Vaccines are expected to prevent or decrease sickness after monkeypox infection because they are effective at preventing illness before exposure. Indicated for post-exposure prophylaxis in high-risk persons, they can be considered on an individual basis in those with intermediate-risk exposures. Vaccination is recommended within 4 days of exposure to avoid illness. Immunization between 4 and 14 days after exposure may reduce illness occurrence but does not prevent disease. Requests should be sent to state or territory health authorities [71]. The CDC Emergency Operations Center (phone: 1-770-488-7100) is available for consultations on vaccination use [72]. Vaccines for post-exposure prophylaxis are available through the CDC’s Strategic National Stockpile. Some public health officials are employing a broader strategy for post-exposure prophylaxis for the 2022 outbreak event, delivering vaccines to persons who are at a higher risk of recent monkeypox exposure, even if they have not been exposed to someone with confirmed monkeypox [73]. 

### 8.3. Vaccinia Virus Vaccine

New York City’s Board of Health Adults: Percutaneous scarification with live antigen solution 0.0025 mL (a droplet of vaccine) was administered subcutaneously (through 15 rapid needle punctures) to the arm. If a severe reaction is not seen 6 to 8 days after vaccination, check immunization methods and repeat vaccination with vaccine from a new vial or vaccine lot, if available. If the initial attempt did not result in a major reaction, consult the CDC, state or local health department, or another vial or lot of the vaccine before delivering a second dose. Patients who continue to be at high risk of monkeypox exposure may require a booster dose every three years. For the booster shot, make 15 needle punctures. Do not revaccinate because the cutaneous response may be decreased [74,75,76].

### 8.4. Jynneos

Approved for patients 18 years of age and older who are at high risk for infection for preventing smallpox and monkeypox disease. An EA-IND protocol is being prepared by the CDC for use in pediatric populations. The only vaccine used in current vaccination campaigns for the 2022 monkeypox outbreak response is the Jynneos vaccine, though supply is constrained by the necessity of preparation of a live, non-replicating virus (vaccinia). Two subcutaneous injections, separated by four weeks, are necessary for vaccination completion; two weeks after the second dose, vaccination is deemed complete [21,55]. 

Adults: 0.5 mL subcutaneously for 2 doses given 4 weeks apart with modified Vaccinia Ankara Suspension. Post-exposure prevention 19–55: Adults: 0.5 mL subcutaneously for 2 doses given 4 weeks apart with modified Vaccinia Ankara Suspension. To stop the disease from developing, administer within 4 days of the exposure date. Vaccination may lessen disease symptoms if given between 4 and 14 days after exposure, but it cannot prevent disease. Consider revaccinating if it has been more than 3 years since your last vaccination [68,77,78,79,80].

## 9. Conclusions

It is important for clinicians to be on the lookout for rashes that could be compatible with monkeypox, especially in patients who have established high-risk epidemiologic markers (i.e., contact with someone with similar rash or confirmed monkeypox diagnosis, close contact with people in a social network with monkeypox activity, history of recent international travel to country with confirmed cases). Reports of possible cases of monkeypox should be made to the appropriate hospital as well as to the local and state public health authorities (to facilitate necessary reporting and further diagnostic and management steps). To limit the spread of monkeypox in the community, rapid identification, diagnosis, isolation and management of affected patients in the sporadic and endemic regions are required. In addition, health care professionals must be educated to identify and manage cases of monkeypox. Targeted health promotion through community involvement sensitively supports enhanced testing and education in populations at risk is needed. 

## Figures and Tables

**Table 1:** Global high burden countries.

Country	Cases
United States	25,340
Brazil	7445
Spain	7122
France	3970
Germany	3607
United Kingdom	3585
Peru	2423
Colombia	1653
Canada	1389
Mexico	1367
The Netherlands	1221
Portugal	917
Italy	846
Chile	842
Belgium	757
Switzerland	513
Argentina	326
Austria	309
Nigeria	277
Israel	250
Democratic Republic of the Congo	195
Sweden	186
Denmark	184
Poland	182
Ireland	178
Bolivia	175
Australia	135
Ecuador	120

## Data Availability

Data are contained within the manuscript.
